# Serial changes in the aqueous IL-10 level after intravitreal methotrexate injection as an indicator of primary vitreoretinal lymphoma recurrence

**DOI:** 10.1038/s41598-020-73111-2

**Published:** 2020-10-02

**Authors:** Young Gun Park, Woo-Kyung Park, Rae-Young Kim, Mirinae Kim, Young-Hoon Park

**Affiliations:** 1 Department of Ophthalmology and Visual Science, Seoul St. Mary’s Hospital, College of Medicine, The Catholic University of Korea, 222 Banpo-daero, Seocho-gu, Seoul, Korea; 2 Catholic Institute for Visual Science, Collage of Medicine, The Catholic University of Korea, Seoul, Korea

**Keywords:** Retinal diseases, Vision disorders

## Abstract

Primary vitreoretinal lymphoma (PVRL) often masquerades as other uveitic diseases. We investigated the aqueous cytokine level changes and the effects of intraocular methotrexate (MTX) in patients with PVRL. In this retrospective consecutive case-series study, we reviewed the records of 14 consecutive patients with PVRL treated between 2018 and 2020. The concentrations of interleukin (IL)-2, IL-6, IL-10, IL-12, IL-17, interferon (IFN)-γ, and tumor necrosis factor (TNF)-α were determined at baseline and several time points after intravitreal MTX injections during follow-up. Markedly elevated IL-10 levels and a higher IL-10/IL-6 ratio were found in patients with PVRL. The aqueous levels of IL-10, IL-12, and TNF-α, and the IL-10/IL-6 ratio significantly decreased at 1 month after intravitreal MTX therapy onset compared with the baseline values (*P* = 0.001, 0.002, 0.001, and 0.001, respectively). The mean duration to normalized IL-10 levels was 1.17 ± 0.4 months. Where serially recorded IL-10 levels were available, regular intravitreal MTX treatment was associated with rapid reduction in IL-10 levels, while elevated IL-10 level was associated with disease recurrence. Elevated IL-10 levels and high IL-10/IL-6 ratio may aid in the diagnosis of PVRL. Aqueous IL-10 level monitoring can help assess the therapeutic response and indicate disease recurrence.

## Introduction

Primary vitreoretinal lymphoma (PVRL) is a very rare disease with only approximately 380 cases diagnosed every year in the United States, but its incidence has been gradually increasing in the recent years^1,2^. The survival prognosis of patients with PVRL is determined based on disease extension to the central nervous system (CNS) within 3 years^3^. PVRL is a typical masquerade syndrome that presents with nonspecific symptoms, such as blurred vision and floaters, often being confounded with other uveitis types for a long time and treated with corticosteroids.

The gold standard for PVRL diagnosis is cytological analysis, usually made by examination of the vitreous after diagnostic vitrectomy or by retinal biopsy. However, this method poses difficulties due to problems with specimen preservation and fragility of the lymphoma cells. False-negative cytology results have been reported in nearly 30% of cases or more^4–6^.

Recently, many studies have reported on less invasive methods of diagnosing PVRL and monitoring the therapeutic response through measurement of the intraocular cytokine levels in the anterior chamber. Interleukin (IL)-10, a cytokine produced by malignant B cells, has been recognized as a valuable cytokine in lymphoma; it is related to B-cell antibody production and its intraocular levels reflect the rate of malignancy of the lymphoma. On the other hand, IL-6 levels are elevated in eyes with inflammatory uveitis, but not in eyes with neoplastic disease. Multiple studies have shown that an IL-10/IL-6 ratio greater than one or increased IL-10 level of more than 150 pg/mL supports the diagnosis of lymphoma^7,8^. Systemic chemotherapy, such as that with intravenous methotrexate (MTX), is the first-line treatment; however, systemic administration is associated with severe side effects^9^. In order to avoid these problems, intravitreal MTX injections have been recommended in cases of PVRL with favorable outcomes and less, acceptable side effects. Still, the guidelines on optimal follow-up for intravitreal MTX injections remain unclear.

In the present study, we evaluated the serial changes in the cytokine profile of CD4-positive T-helper clones (Th1/Th2), including IL-6, IL-10, interferon-gamma (IFN-γ), IL-2, IL-12, IL-17, and tumor necrosis factor-alpha (TNF-α), in the aqueous humor of patients with PVRL treated with intravitreal MTX injections and we analyzed the relation of these changes with the patients’ clinical course and the recurrence of PVRL.

## Results

A total of 14 patients, six male and eight female patients, who were diagnosed with PVRL were enrolled. The mean age (mean ± SD) at diagnosis was 67.0 ± 9.28 years and the mean follow-up period was 18.35 ± 8.23 months. Eight patients (57.1%) had CNS involvement and 9 (64.3%) had involvement of the fellow eyes. The mean number of received intravitreal MTX injections was 18.71 ± 8.23. Eight of the 14 patients underwent pars plana vitrectomy for cytological confirmation; PVRL was confirmed by cytology only in six patients (Table 1).

**Table 1:** Patients’ baseline demographics, clinical findings, and disease course.

No.	Age (years)	Sex	OD/OS	FE involvement	CNS involvement	Systemic CTx	Disease confirmed	IL-6	IL-10	IL-10/IL-6 ratio	No. of MTX injections	F/U duration	Recurrence status
1	70	F	OS	+	+	+	+	121.1	327.1	2.70	21	14	+
2	76	F	OS	+			+	146.3	202.9	1.39	24	38	
3	75	M	OD				−	10.3	9452	917.67	16	13	
4	55	M	OD	+	+	+	−	554.7	16,107	29.04	27	15	+
5	63	F	OS	+	+	+	+	54.9	5087	92.66	22	44	+
6	55	F	OD	+			+	5.5	613.4	111.53	17	34	
7	58	F	OD		+	+	−	12.2	2160	177.05	9	10	
8	79	F	OS		+	+	+	32.9	344	10.46	4	8	
9	67	F	OD	+			Unsatisfactory	288.5	16,653	57.72	25	11	+
10	72	M	OS	+	+	+	+	260.9	361.7	1.39	21	14	+
11	62	M	OS				−	8.9	4161	467.53	31	13	+
12	82	M	OS		+	− (refused)	−	230	23,882	103.83	28	31	
13	72	F	OS				−	63.8	645	10.11	5	7	
14	52	M	OD	+	+	+	Unsatisfactory	235.9	14,218	60.27	12	5	

*OD* right eye, *OS* left eye, *FE* fellow eye, *CNS* central nervous system, *CTx* chemotherapy, *IL* interleukin, *MTX* methotrexate, *F/U* follow-up.

### Changes in aqueous cytokine levels at 1 month

The mean levels of all measured cytokines, except IL-6, were reduced at 1 month after initiation of intravitreal MTX treatment. The mean IL-10/IL-6 ratio also decreased from 141.23 ± 244.71 to 0.98 ± 1.45. The changes from baseline were significant for the levels of IL-10, IL-12, and TNF-α, and for the IL-10/IL-6 ratio (*P* = 0.001, 0.002, 0.001, and 0.001, respectively; Table 2). The mean duration to normalized IL-10 levels was 1.17 ± 0.4 months. The mean number of MTX injections required to normalize IL-10 levels was 8.54 ± 1.92.

**Table 2:** Changes in the aqueous cytokine levels in patients with PVRL after 1-month intravitreal MTX treatment.

	Baseline	After 1 month of treatment	*P*-value
IFN-γ (pg/mL)	8.17 ± 14.23	0.55 ± 0.67	0.015
IL-2 (pg/mL)	1.55 ± 0.82	1.07 ± 0.63	0.099
IL-6 (pg/mL)	139.21 ± 152.44	957.54 ± 2334.34	0.552
IL-10 (pg/mL)	6592.41 ± 7641.88	21.98 ± 1.84	0.001*
IL-12 (pg/mL)	20.85 ± 24.87	1.84 ± 2.53	0.002*
IL-17 (pg/mL)	9.52 ± 15.93	3.82 ± 1.73	0.530
TNF-α (pg/mL)	27.45 ± 25.33	2.92 ± 3.05	0.001*
IL-10/IL-6 ratio	141.23 ± 244.71	0.98 ± 1.45	0.001*

Data are presented as mean ± standard deviation. PVRL, primary vitreoretinal lymphoma.

*MTX* methotrexate, *IFN-γ* interferon gamma, *IL* interleukin, *TNF-α* tumor necrosis factor alpha.

*P < 0.007.

### Changes in aqueous cytokine levels in patients with disease recurrence

Even with continued treatment, six patients experienced disease recurrence during the follow-up period. At the time of recurrence, the mean cytokine levels, except that of IL-6, were increased compared to those measured at 1 month after MTX treatment start. After 1 month of retreatment with intravitreal MTX injections, the mean cytokine levels reduced again (Table 3).

**Table 3:** Changes in aqueous cytokine levels in patients with disease recurrence.

	At recurrence (pg/mL)							After 1-month retreatment (pg/mL)						
	IFN-γ	IL-2	IL-6	IL-10	IL-12	IL-17	TNF-α	IFN-γ	IL-2	IL-6	IL-10	IL-12	IL-17	TNF-α
Case 1	1.1	< 1.0	35.8	370.9	4.3	4.1	5.6	N/A	N/A	6.5	1	N/A	3.9	1.3
Case 4	N/A	1.3	8.9	917.2	9.2	4.3	1.6	N/A	1.7	13.7	1	N/A	4.9	1.4
Case 5	N/A	1.5	13.3	2051	N/A	5.5	2	N/A	1.7	1.8	2.5	N/A	8.2	N/A
Case 9	N/A	1.9	181.2	2615	N/A	4.7	14.1	N/A	1.6	232.9	53.5	N/A	6.5	1.7
Case 10	N/A	1.2	176.7	267.5	4.3	2.8	4.9	N/A	1.6	39.6	86.3	N/A	4.5	2.2
Case 11	3.8	2.5	22.8	9149	10.9	4.5	9.2	N/A	1.1	62.2	1.3	N/A	3.6	2.1

*IFN-γ* interferon gamma, *IL* interleukin, *TNF-a* tumor necrosis factor alpha, *N/A* not available (less than 1.0 pg/mL).

### Representative cases with disease recurrence

**Figure 1:**
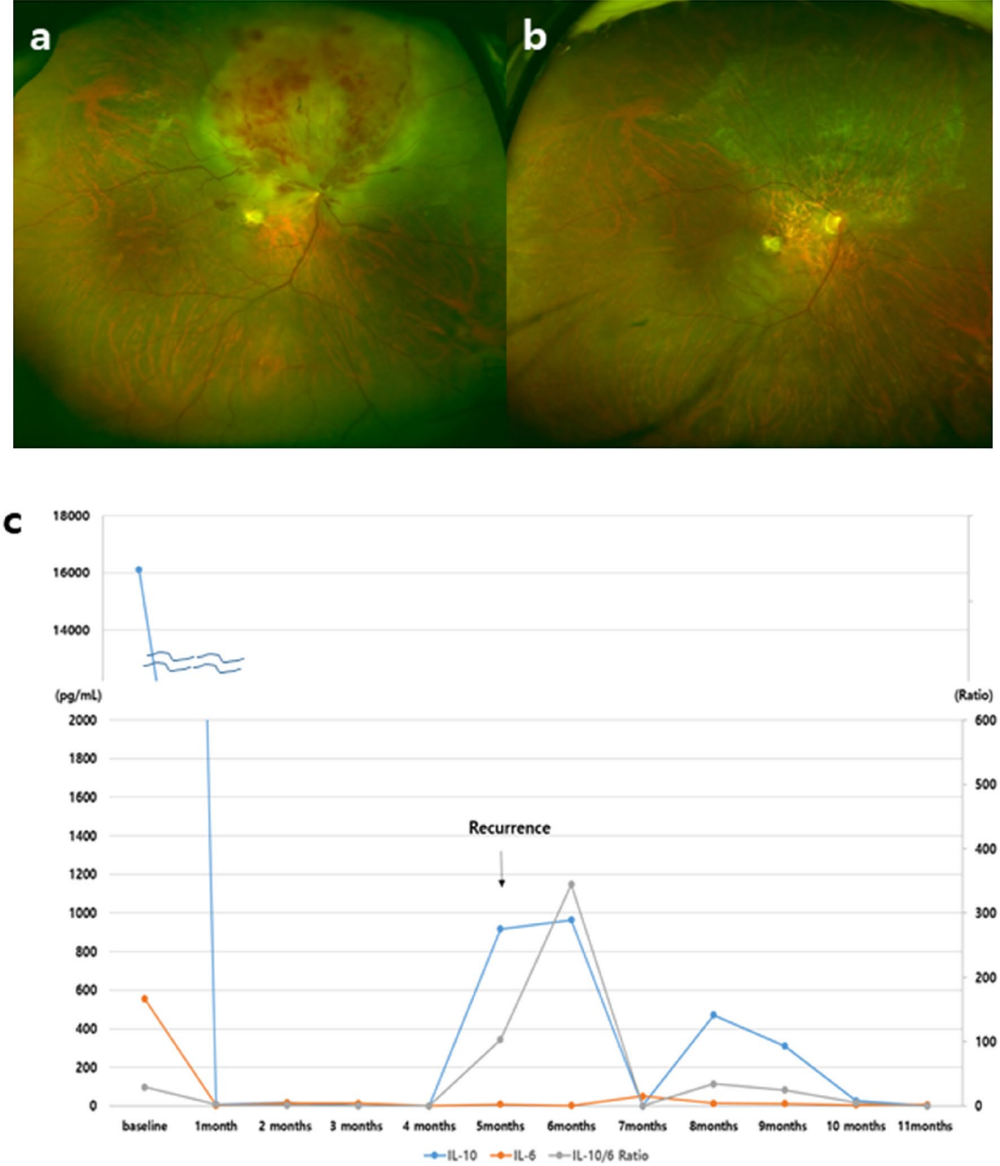
Case 4. (**a**, **b**) Fundus photographs at baseline and at 6 months. (**c**) Monthly intraocular interleukin-10 (IL-10) and IL-6 measurements during follow-up. A rapid decrease in the IL-10 level from 16,107 to 80.6 pg/mL was noted after the onset of intravitreal methotrexate injections. The increase in the IL-10 level at 5 months corresponded to intraocular lymphoma recurrence.

*Case 4* (Fig. 1). This was a 55-year-old male patient with an IL-10 level of 16,107 pg/mL and an IL-10/IL-6 ratio of 29.04 at baseline. He presented with severe retinal infiltration and we decided to monitor his cytokine levels monthly. Positron emission tomography-computed tomography and magnetic resonance imaging did not show any brain involvement. After initiation of intravitreal MTX treatment, the IL-10 and IL-6 levels dramatically decreased from 16,107 pg/mL and 554.7 pg/mL to 8.6 pg/mL and 3.5 pg/mL, respectively. The IL-10/IL-6 ratio also decreased from 29.04 to 2.46 and was lower than 1 after 3 months. Five months after therapy start, the IL-10 level increased to 917.2 pg/mL and the IL-10/IL-6 ratio also increased to 344.46. The patient was re-evaluated and was diagnosed with large B-cell lymphoma in the nasal cavity and cervical lymph nodes. Retreatment with systemic and intravitreal chemotherapy was administered. A linear decrease in the aqueous IL-10 level was observed during the treatment, with the level becoming undetectable after 1 month of treatment, as is maintained to present (12 months later).

**Figure 2:**
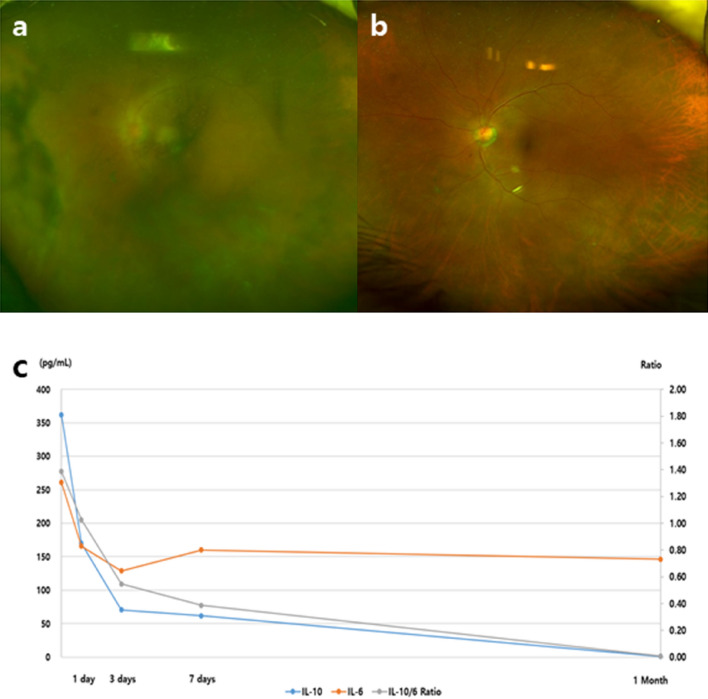
Case 10. (**a**, **b**) Fundus photographs at baseline and at 4 months. (**c**) Intraocular interleukin-10 (IL-10) and IL-6 measurements after intravitreal methotrexate injections within 1 month. The initial IL-10 and IL-6 levels were 361.7 pg/mL and 260.9 pg/mL, respectively. A linear decrease in the IL-10 level was observed within 3 days and became undetectable at 1 month. The IL-10/IL-6 ratio also decreased over time.

*Case 10* (Fig. 2). This was a 72-year-old male patient in whom PVRL was confirmed by diagnostic vitrectomy and who had CNS involvement. The initial mean intraocular IL-10 level was 361.7 pg/mL; it decreased to 170.1 pg/mL at 24 h and 70.6 pg/mL at 72 h after MTX injection, and became undetectable at 1 month after treatment onset. The initial IL-10/IL-6 ratio was 1.39, and a similar decrease was observed until it reached 0.01 over time. The patient was followed up for 4 months and had received 12 MTX injections when his IL-10 level increased to 267.5 ng/L and the IL-10/IL-6 ratio increased to 1.51. At the same time, recurrence was detected clinically with reappearance of vitritis. The patient received a new intravitreal MTX injection that improved the vitritis and normalized the IL-10 level by the 1-month visit. He has been under observation for 8 months.

## Discussion

PVRL is difficult to diagnose because it often masquerades as inflammatory or infective posterior uveitis, which can lead to delays in the correct diagnosis of a disease that is ultimately life-threatening. Cytological confirmation is limited to only 30–45% of PVRL cases. A delay in diagnosis is related with poor visual outcomes and increased mortality, because 40–60% of patients with PVRL develop CNS involvement within a mean of 8–29 months^10,11^. Thus, development of methods for earlier and more accurate diagnosis is of utmost importance.

There has been an increasing trend emphasizing the role of molecular diagnostic tests. The most promising methods are immunophenotyping (including immunoglobulin heavy chain rearrangement), molecular analysis (MYD88 and gene rearrangement), and cytokine level analysis^12,13^. Recently, the increase in IL-10 levels or IL-10/IL-6 ratio in the aqueous humor has been proposed as a new diagnostic tool^14–16^. Pochat-Cotilloux et al. measured the concentrations of IL-10 and IL-6 in both the vitreous and the anterior chamber and demonstrated that a ratio higher than one had a sensitivity of 93% and specificity of 100% for diagnosing PVRL^17^. The cut-off value for IL-10 in the anterior chamber was 30 pg/mL and the overall diagnostic sensitivity and specificity were calculated above 78% and 97%, respectively. The authors demonstrated that the use of a cut-off value for IL-10 concentration and IL-10/IL-6 ratio could help for an easier diagnosis of PVRL. In this study, we used a cut-off value of 150 pg/mL for IL-10 or a IL-10/IL-6 ratio > 1 as diagnostic criteria for PVRL, based on previous reports^13,16,18^.

In addition to timely diagnosis, monitoring of treatment efficacy and detecting recurrence is also paramount^19^. Visual acuity and vitreous transparency have been the common criteria for assessing treatment efficacy and disease recurrence^20–22^. In this study, the serial changes in the IL-10 levels in the anterior chamber allowed an objective and less aggressive assessment for disease recurrence. Because anterior chamber paracentesis is relatively simple compared with vitreous biopsy, we propose that it be used as a less invasive test to detect recurrence. In our study, normalization of the IL-10 level was achieved in all cases 1 month after the first MTX injection. At disease recurrence, the aqueous levels of IL-10 rapidly increased and again decreased after intravitreal MTX injections in all six patients. This could simply reflect the fact that IL-10 is a growth and differentiation factor for B lymphocytes, and its level is directly associated with the number of malignant cells.

Furthermore, other cytokines, including IL-12, and TNF-α, were also significantly decreased at 1 month after intravitreal MTX therapy onset. Th1 cytokines including IL-12 activate cytotoxic T lymphocytes and natural killer cells, which play critical roles in antitumor immune responses. The levels of these cytokines are essential both for the intrinsic antitumor response as well as for effective immunotherapy^23,24^. TNF-α is also involved in pathological processes, such as chronic inflammation and malignant disease, and may be a target molecule for cancer therapy^25,26^. However, the levels of these cytokines at baseline are too low (less than 1 pg/mL) in some patients, and are therefore difficult to detect. Moreover, in this study, most of the cytokines, except for IL-10, were not detected at the time of disease recurrence.

Therefore, this study provided direct evidence of the role of IL-10 in PVRL control. Based on our results, the increase in the IL-10 level could be useful not only for diagnosing PVRL but also for monitoring during follow-up. However, further research is needed to elucidate all possible influencing factors.

The initial evaluation of Case 4 in this study showed no systemic involvement. After 5 months, the aqueous IL-10 level rapidly increased from 0.01 to 917.2 pg/mL. Re-evaluation revealed malignant lymphoma in the nasal cavity that was confirmed by histological examination. The patient was treated with systemic chemotherapy and intravitreal MTX injections and showed a noticeable improvement. This case demonstrated that the changes in the IL-10 levels can aid the diagnosis of recurrence or spread of the disease.

The most critical thing is that early diagnosis and accessible monitoring results in a better prognosis. The efficacy of IL-10 concentration and the IL-10/IL-6 ratio compared with that of other significant markers, such as MYD88 and gene rearrangement, is still being verified, but these might ultimately prove more precise and accessible markers. Furthermore, it is possible that the serial changes in the IL-10 levels in the aqueous humor may be useful for determining the treatment response or suggesting disease recurrence. Although repeated IL-10 measurements remain a burden, this will reduce the number of follow-up visits for patients.

Our study had several significant limitations. It was a retrospective study that included only a relatively small sample without controls, and had a relatively short follow-up. However, randomized clinical trials are difficult to perform due to the low incidence of PVRL.

In conclusion, we demonstrated that elevated IL-10 levels and a ratio of IL-10/IL-6 > 1 can be used for diagnosing PVRL. At disease recurrence, the concentration of IL-10 rapidly increased and again decreased after beginning treatment. Therefore, we may be able to modify the treatment protocols based on the changes in the IL-10 levels. Nonetheless, further large clinical trials are recommended to confirm the diagnostic and monitoring values of IL-10 levels and establish new treatment protocols for recurrent PVRL.

## Methods

The study was approved by the Ethics Committee of Seoul St. Mary’s Hospital, The Catholic University of Korea. The requirement for informed patient consent was waived due to the retrospective nature of the study. All procedures were conducted in accordance with the Declaration of Helsinki (1964) and its later amendments.

This was a retrospective consecutive case-series study in patients diagnosed with PVRL who were treated at the Department of Ophthalmology of Seoul St. Mary’s Hospital in Seoul, Korea, between January 2018 and January 2020. We reviewed their medical records and analyzed the recorded cytokine levels and treatment course.

We initially diagnosed PVRL according to typical clinical features and cytokine levels. The utility of IL-10:IL-6 ratios and total IL-10 concentration in combination with the distinct clinical pattern can provide a more accurate diagnosis, even if histopathological confirmation is lacking. Thus, PVRL was diagnosed based on an IL-10/IL-6 ratio greater than one or increased intraocular IL-10 level of more than 150 pg/mL. Patients with thick vitreous opacities on fundus examination underwent diagnostic 25-gauge pars plana vitrectomy. Cytological analysis was performed using samples of at least 0.4 mL of undiluted or 0.8–1.0 mL of diluted vitreous.

MTX injections were administered according to the therapeutic regimen^27^. Initially, 400 μg/0.1 mL of MTX was injected intravitreally twice per week for 1 month, followed by weekly injections for 1 month, and then followed by monthly injections for 1 year as maintenance treatments. At the doctor’s discretion, 0.1 cc of aqueous humor were sampled to determine the intraocular cytokine concentration, including IL-6, IL-10, IFN-γ, IL-2, IL-12, IL-17, and TNF-α.

We used the MILLIPLEX MAP Human cytokine magnetic bead panel (Merck Millipore Corporation, Billerica, MA, USA) to quantify cytokine concentrations. Samples including standards and quality controls were incubated with magnetic beads coated with antibodies against individual cytokines and analyzed in duplicate according to the manufacturer's instructions. Each sample was analyzed using a LUMINEX MAGPIX system (Luminex Corp., Austin, TX, USA).

During the follow-up, recurrence was detected based on visual acuity decrease and accompanying vitritis. These patients received new intravitreal MTX injections at the time of aqueous humor sampling. Injections were stopped when the lymphoma was no longer clinically active and close monthly monitoring was continued.

Statistical analysis was performed using SPSS version 18.0 (SPSS, Chicago, IL, USA). Wilcoxon’s signed-rank test was performed for data analysis, and Bonferroni correction was used for multiple comparisons. According to Bonferroni correction, P < 0.007 was considered statistically significant.

## Data Availability

The datasets analyzed during the current study are available from the corresponding authors on reasonable request.
